# Geometrical digital twins of the as-built microstructure of three-leaf stone masonry walls with laser scanning

**DOI:** 10.1038/s41597-023-02417-3

**Published:** 2023-08-10

**Authors:** Savvas Saloustros, Andrea Settimi, Andrea Cabriada Ascencio, Julien Gamerro, Yves Weinand, Katrin Beyer

**Affiliations:** 1 Earthquake Engineering and Structural Dynamics Laboratory (EESD), School of Architecture, Civil and Environmental Engineering (ENAC), Institute of Civil Engineering (IIC), EPFL, Lausanne, 1015 Switzerland; 2 Laboratory for Timber Constructions (IBOIS), School of Architecture, Civil and Environmental Engineering (ENAC), Institute of Civil Engineering (IIC), EPFL, Lausanne, 1015 Switzerland

**Keywords:** Civil engineering, Structural materials

## Abstract

Research on irregular stone masonry walls is hampered by the lack of detailed geometrical models of their internal micro-structure, i.e. the shape and size of each stone and its position within the wall. Without such a geometric digital twin of walls tested in the laboratory, it is difficult to evaluate the accuracy of existing numerical simulation techniques. Here, we describe the generation of geometrical digital twins of three irregular stone masonry walls built in the laboratory. We labelled each stone manually and then obtained the geometry of the individual stones using a portable laser scanning device. With the same device we scanned the wall after the construction of each layer. We then registered the position of each stone in the layer. This paper outlines the methodology for the data acquisition and digital reconstruction and presents the datasets for the walls. The developed geometrical digital twins provide unique information regarding the micro-structure of constructed walls that is key for the development and validation of numerical simulation techniques for stone masonry.

## Background & Summary

The structural behavior of stone masonry walls is directly related to both the mechanical properties of the comprising stones and binding mortar as well as to the geometrical arrangement of these components, termed the micro-structure of the wall, as evidenced by experimental^1–3^ and numerical studies^4–8^. While there are standardized experimental procedures for mechanically characterizing the components of a wall^9–11^, it is still a challenge to determine the structural effect of its micro-structure^8^. This is particularly true for irregular stone masonry walls that do not have a repetitive arrangement of stones and mortar, thus presenting a unique micro-structure that bestows a unique structural behavior (e.g., displacement and strength capacity, stiffness).

Current technical codes^12,13^ consider the effect of wall micro-structure by adopting a wall-typology categorization system. This system defines a certain number of stone masonry categories based on their micro-structure and correlates each category with a range of key mechanical properties. Engineers then assign the investigated masonry typology to one of the defined categories to determine the relevant mechanical properties. While this approach is a first step towards estimating the mechanical properties of stone masonry walls, it has two important limitations. First, the fixed number of categorized typologies does not adequately reflect the wide variety of existing masonry micro-structures. This makes category assignment a subjective decision, as masonry walls commonly display construction characteristics from more than one of the defined categories. Second, even within the same masonry typology, the listed ranges for the mechanical properties are wide enough to require the consideration of numerous uncertainties during structural analysis as evidenced experimentally^3^ and numerically^5^. The Masonry Quality Index (MQI)^14^ is an alternative to the wall-typology categorization system for the characterization of stone masonry walls. It is based on assessing seven morphological characteristics of the wall as fulfilled, partially-fulfilled or not fulfilled. A numerical value is given to each of them based on its fulfillment category. MQI is computed as the weighted sum of these characteristics and has shown to correlate well with strength (compressive, shear) and stiffness values of stone masonry. The assessed masonry characteristics are the conservation state, the stone shape and dimensions, the wall leaf connections, the mortar quality and the horizontality and verticality of bed and head joints, respectively. This information is derived from visual inspection and 2D images of the wall, while indices related with the 3D geometry (e.g. thickness and shape and distribution of stones in the interior) are currently missing. Overall, these issues make it difficult to accurately predict the structural response of stone masonry walls with varying geometry through the thickness^15–18^.

Numerical simulations offer an alternative way for estimating key engineering parameters and the resulting structural response to a categorization-based system. The accuracy of these simulations relies on the choice of numerical approximation approach, the realistic representation of the mechanical response (i.e. constitutive behavior and input mechanical properties) and of the geometry (i.e. global structure and micro-structure). Thus, even though numerical simulations techniques^19,20^ and testing^21^ have advanced resulting in an accurate numerical approximation and mechanical characterization of the materials, respectively, the lack of a detailed knowledge of the micro-structure of experimentally tested walls–a geometrical digital twin–represents today a bottleneck in the validation of numerical simulation approaches for irregular stone masonry. The absence of a geometrical digital twin of a stone masonry wall tested in the lab increases the uncertainties involved in the simulation and hampers a fair comparison between numerical and experimental results. For single-leaf walls with rather prismatic stones, the microstructure can be estimated from an image of the wall (e.g.^6,7,22^). For walls with multi-leaves, images of the outer leaves do not provide complete information about the microstructure. It is largely due to this that, until today, we lack a validated numerical simulation procedure for irregular stone masonry walls with several leaves, which is one of the most vulnerable masonry typologies when subjected to seismic loading.

There are two current best approaches for acquiring the micro-structure of existing stone masonry walls: non-destructive tests^23–29^ and computer vision techniques^6,7,22,30^. Non-destructive techniques, such as sonic tomography and ground penetrating radar, aim to identify the internal morphology of existing stone masonry walls and combine this information with external visual inspection (e.g. images, laser-scanning survey). While these techniques can identify some key elements of stone masonry walls (e.g. number and thickness of layers through the wall section), they still cannot accurately represent the internal microstructure of the wall. Alternatively, computer vision techniques can transform an image of a stone masonry wall to a numerical model with the accuracy defined by the image resolution (e.g. pixel size) and the level of detail of the analysis method (e.g. mesh size in the numerical model). Nonetheless, they do not have any information on the internal morphology of the wall. A geometrical digital twinning pipeline based on images that can generate the internal morphology has been recently presented in^31^. This technique allows a largely automated reconstruction of the geometric digital twin of stone masonry walls with any number of layers, using as input only RGB images of the stones and RGB images taken during the construction phase of the wall. This method was not yet available when generating the geometric digital twins of this dataset and was in the original paper only be applied to a prototype wall of few layers. Following the same methodology, geometrical digital twins of six double-leaf walls with small inner core were recently presented in^32^. These were used for the validation of an automated sonic tomography system. As a result, numerical simulations of real-stone masonry walls tested in the laboratory are restricted to single-leaf regular walls or require assumptions regarding variations of the wall’s morphology through the thickness.

In this paper, we present the generation of the geometrical digital twins corresponding to the as-built geometry of three irregular stone masonry walls using a pipeline based on laser scanning. We describe the pipeline such that it can be reproduced in any structural engineering laboratory with the required equipment. We also provide the generated datasets and to the best of our knowledge, this work provides the first dataset on the microstructure of real three-leaf irregular stone masonry walls with mortar–a novel contribution to the field of structural analysis of this construction typology.

## Methods

### Physical specimen construction

We present in this paper the geometric digital twins of three stone masonry walls (denoted hereafter as SW1, SW2 and SW3), constructed by two experienced masons at the Structural Engineering Laboratory of EPFL. The walls were constructed as part of an experimental campaign for investigating the in-plane seismic response of historic stone masonry walls.

**Fig. 1:**
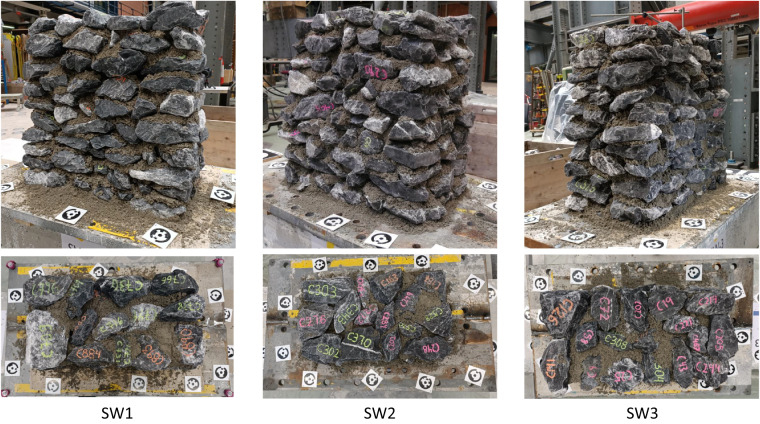
The three rubble stone masonry walls (top) and representative sections with the distribution of the stones through the thickness (bottom) used in this study. From left to right, the walls are labeled SW1, SW2, SW3. For scale purposes, consider that the side of the square markers is 80 *mm* and the diameter of the black circles 65 *mm*.

The construction typology is rubble stone masonry with irregularly shaped limestone units and lime-based mortar. Figure 1 presents the three walls and a corresponding section of each that reveals the three-leaf composition without the use of through stones. The approximate dimensions of the walls are 700 *mm* × 700 *mm* × 400 *mm* (height × length × width). The construction process consisted of the layer-by-layer placement of stones, with no special care for the horizontal and vertical alignment of bed and head joints.

### Geometrical digital twinning pipeline

The pipeline used for the geometrical digital twinning of the stone masonry walls consists of three stages: (i) laser scanning of stones, (ii) laser scanning during construction of walls, and (iii) digital reconstruction. In the following, we present the equipment and the activities involved at each stage.

#### Equipment

We obtained the geometry of individual stones and walls through 3D laser scanning (LiDAR). We used the handheld laser scanner Freestyle Faro 2 to produce an RGB point cloud. LiDAR was selected due to the efficient and fast generation of scaled dense point clouds of the surveyed objects.

During scanning, we maintained a distance of approximately 0.5–1.0 *m* between the handheld LiDAR and the scanned objects (stones and walls). To reduce the amount of collected data, we set the maximum range to a radius of 1.5 *m* to avoid scanning any surfaces beyond this distance. According to the manufacturer, the scanner has a 3D point accuracy of less than 0.5 *mm*. This means that the scans cannot capture any geometrical feature of the stone surface below 0.5 *mm*. For more information on the used laser scan performance specifications, we refer to the manufacturer’s manual^33,34^.

#### Scanning of the stones

**Fig. 2:**
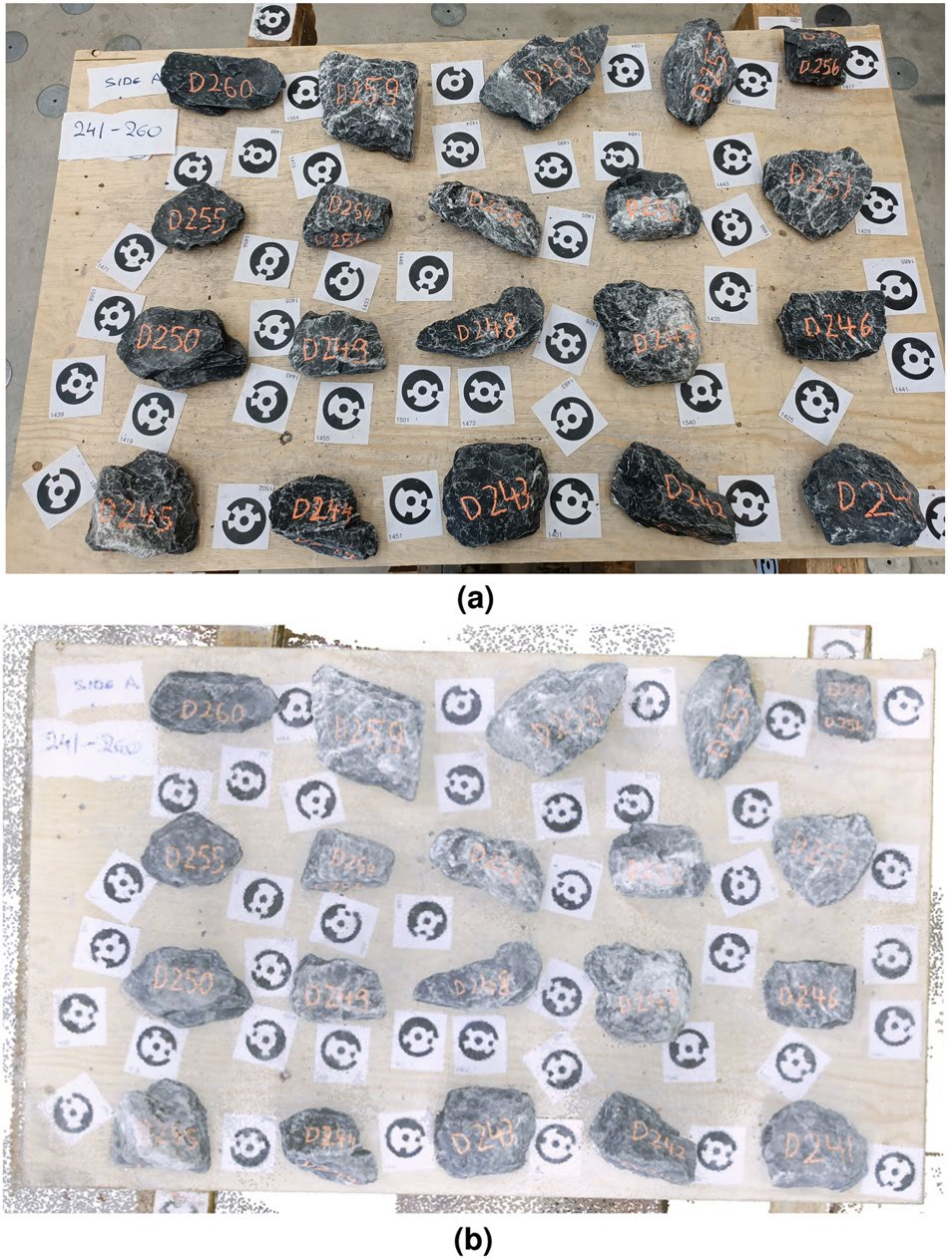
Stone-scanning: (**a**) A batch of 20 stones prepared for scanning and (**b**) top view of the corresponding point cloud. The individual markers used for ensuring well-placed scans can be seen between the stones. For scale purposes, consider that the short side of the rectangular markers is 80 *mm* and the diameter of the black circles 65 *mm*.

To avoid interrupting the wall construction process, we scanned the stones prior to construction. The stones were delivered in bags of 1 *m*^3^. Each bag included approximately 900 stones with maximum dimensions between 50–400 *mm*. Smaller stones (i.e., stone chips) were occasionally used by the masons to fill gaps, but they were not scanned. In total, we scanned four bags corresponding to 3500 stones using the following procedure:

1. *Surface preparation*: Remove dust or broken pieces from each stone using water or air pressure.
2. *Stone labeling*: Label each stone. The label consisted of a letter (A-D) for the bag and a number. For instance, “C425” is the 425th stone scanned from bag C. To ensure that the label is visible during the construction process, labels were written at least on two sides of the stones.
3. *Stone scanning*: Place stones in batches of 20 on the scanning surface. The stones were distributed into 4 rows and 5 columns starting from the lower right corner and increasing in order (according to their label) from right to left and from bottom to top (Fig. 2). Unique numbered markers (see Fig. 2) were placed in-between the stones to ensure well-placed scans (i.e. correct registration to the same coordinate system). After scanning the first side of the stones, all stones were flipped and the second side was scanned. Scanning of a batch of 20 stones lasted in average 10 minutes, which included also the data check and transfer to a personal computer.

#### Scanning of the wall

**Fig. 3:**
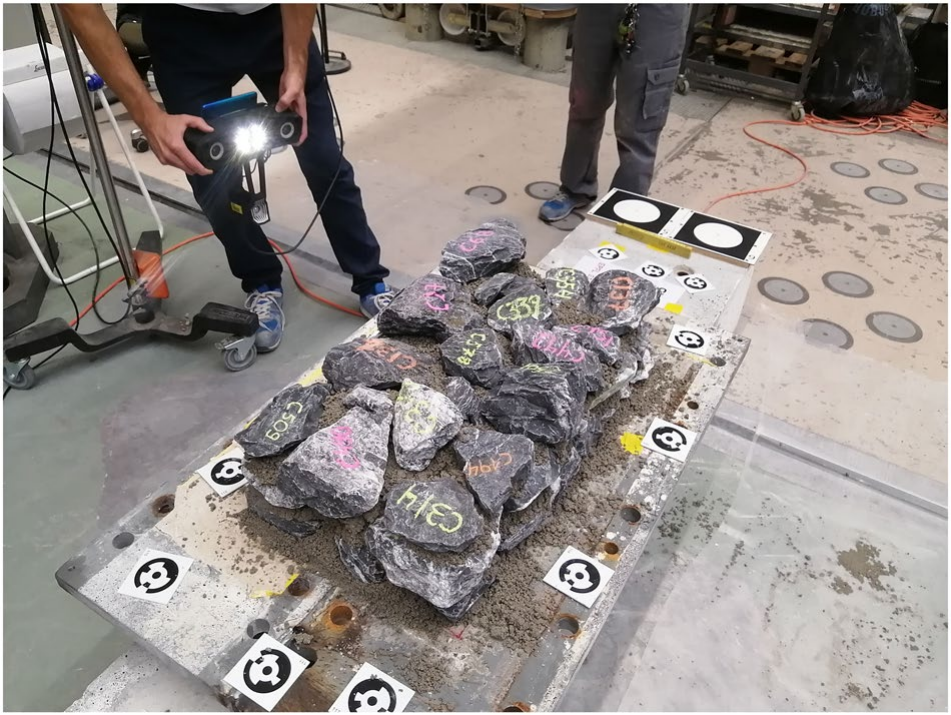
Scanning the wall after placing a new layer of stones. The two out circular targets used in the digital reconstruction procedure can be seen at one end of the foundation. The same targets were placed at the other end of the foundation (not shown here).

As can be seen in Fig. 1, there are several stones at the interior that are not visible from the outside of the wall. To capture the location of these internal stones in our digital twins, we scanned each wall at several stages during the construction. In general, we paused the construction and performed a new scan each time a new layer of stones was placed on top of an existing one (i.e. when a new stone covered a previously placed stone). To reduce the chance of a poor quality scan (due to human error or equipment malfunction), we performed two scans per new layer by walking once around the wall (see Fig. 3). Each scan included the newly constructed part of the wall and four fixed targets placed on the foundation at the two wall sides. The time of each scan increased with increasing height, as the distance between the fixed targets and the new layer of stones increased. For all walls, the scans lasted between 2 and 5 minutes, corresponding to approximately 180 *s*/*m*^2^.

**Fig. 4:**
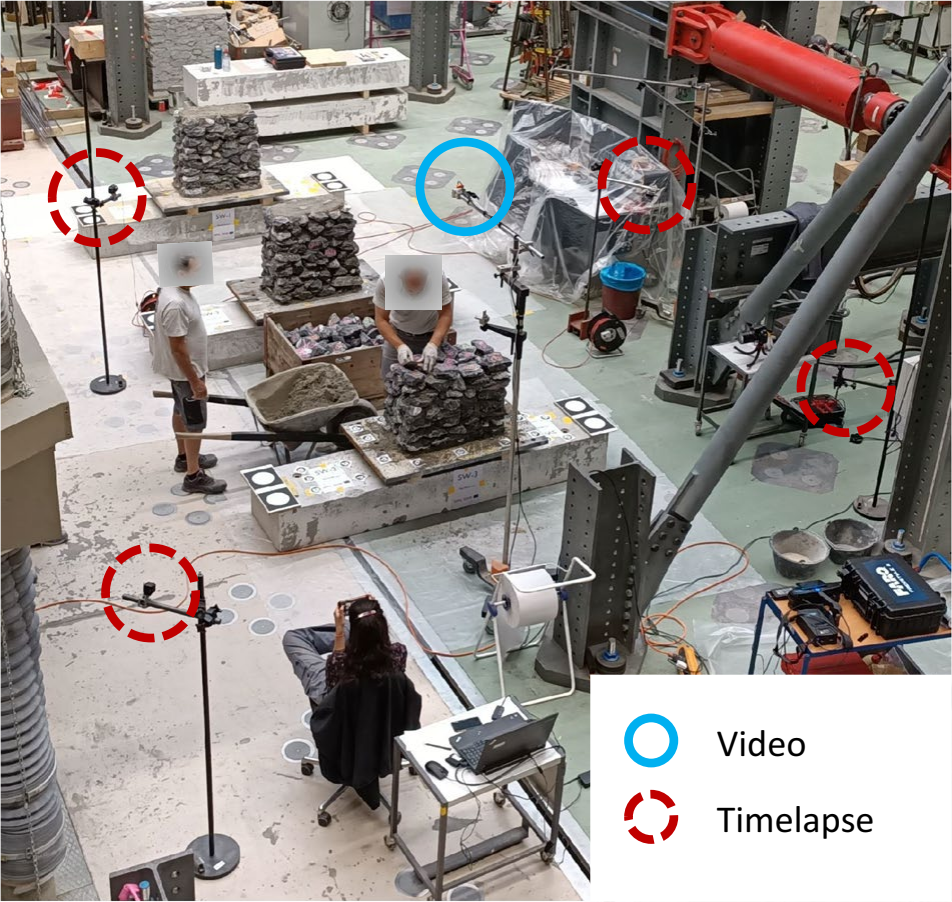
Camera set-up during wall construction.

To identify the location of the stones within the wall using the labels on the stones, several cameras recorded the entire construction process. One camera was placed at the top of the wall and four others were placed at the four corners surrounding the wall (Fig. 4). The top camera recorded a video, while the rest of the cameras captured time-lapse photos (generally every 5”). Additionally, after each scan, photos were taken manually of the top and sides of the walls. The identification of the location of a stone relied mainly on the video from the top. Data collected with other cameras was redundant.

### Digital reconstruction

#### Digital reconstruction of stones

The first step in the digital twinning pipeline is the digital reconstruction of the stones. As presented in the previous section, the point cloud of the total surface of a stone was obtained through two scans, each corresponding to approximately 60–70% of the total surface of the stone (the rest of the stone was in contact with the scanning surface). Each stone was reconstructed by merging the point cloud corresponding with each scan, as outlined in the following. Post-processing of the point clouds was performed using Rhinoceros3D^35^ with the Cockroach plugin^36^ developed at the IBOIS laboratory at EPFL. For a detailed description of the process, refer to the Cockroach documentation website^37^. The procedure for the stone reconstruction was as follows:

1. *Point cloud cleaning*: Import the point cloud (Fig. 5a) and remove all points not belonging to the stones (Fig. 5b). To do so, segment the scanning surface from the rest of the point cloud using random sample consensus (RANSAC) and manually remove the rest of the points below that surface.

**Fig. 5:**
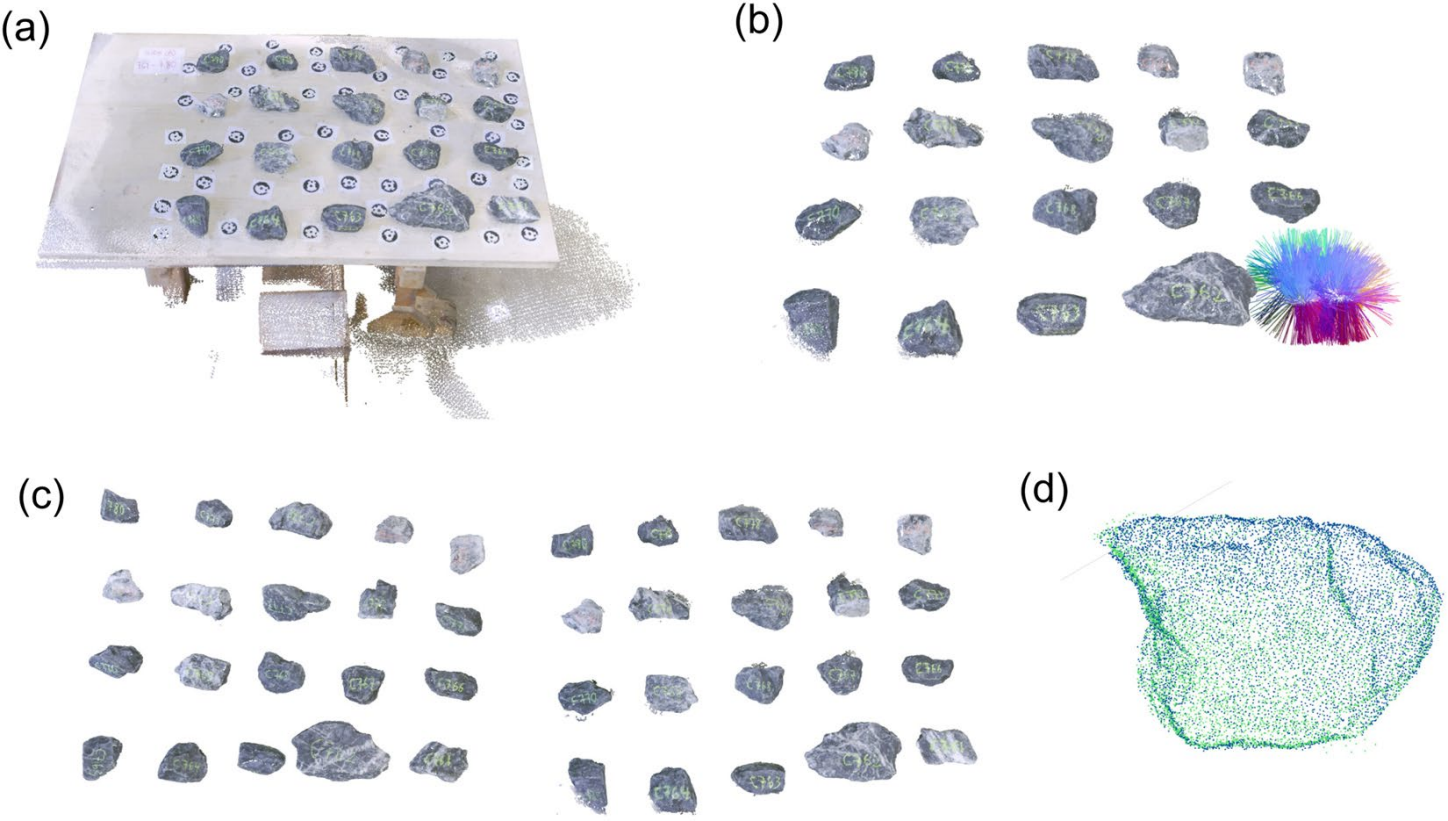
Digital reconstruction of stones. (**a**) Point cloud of one side of the stones before processing. (**b**) Point cloud of stones after removing points not belonging to the stones. The bottom right stone shows the visualization of the computed normals on clusters of points. (**c**) Point clouds of the two sides of the same batch of stones after cleaning, clustering, and normal computation. (**d**) Example registration of the point clouds corresponding to two halves of the same stone. Each point cloud is shown in a different color.
2. *Point cloud down-sampling* (optional): If desired, decrease the point cloud resolution to the desired level. In this work, we used a voxel downsampling procedure to down-sample the point clouds. Voxel downsampling consists in the clustering of points into voxels. The coordinates of all data points inside one voxel were averaged and only this averaged location retained. A voxel size of 2 *mm* was used here, which resulted in average in a decrease of 56% in the number of points per stone’s point cloud (computed considering a batch of 20 stones). Here, we down-sampled in order to reduce the post-processing time.
3. *Clustering of stone point clouds*: Cluster the points belonging to each stone into an individual point cloud.
4. *Computing normals to the surface*: For each stone, compute the normals of the planes made by a selected number of neighboring points (Fig. 5b). Neighbors are selected using a k-NN search, while the direction of the normal is defined through a covariance analysis as described in^38^. The direction of the normals is important for registering the stones, as described in step 6.
5. *Repeating steps 1*–*4 for the second scan of the batch*. Repeat the previous steps for the second scan of the same batch, corresponding to the previously hidden side of the stones. Import the clustered point clouds of the stones into the same CAD environment (Fig. 5c).
6. *Registering the two halves of the stones*: Register the two halves of the stone by minimizing the root-mean-square error (RSME) between the two sets of points. In general, we consider the threshold for the registration to be an RSME value lower than 4 *mm*. (Fig. 5d). This step uses the RANSAC global registration^39^ and ICP local refinement^40^ as implemented in^38^.

The reconstruction of each stone lasted in average 5 minutes. In total, 445 stones were used for the construction of the three walls. We provide the point clouds corresponding to the reconstructed stones as described in the data records section.

#### Digital reconstruction of walls

The first step in the digital reconstruction of the walls requires aligning the scans corresponding to each new layer of the wall. To facilitate this alignment, we placed four circular targets at the two sides of the constructed walls on the foundation before construction that remained throughout the construction process (see Fig. 3). Each scan of a new layer thus included these targets. The procedure for aligning the scans to the first scan of the wall is as follows (see also^37^):

1. *Point-cloud import and cleaning*: Import all point clouds corresponding to the scans of the same wall at different construction stages into a single working file. Manually erase points not belonging to the wall nor to the plane of the four targets (Fig. 6a).

**Fig. 6:**
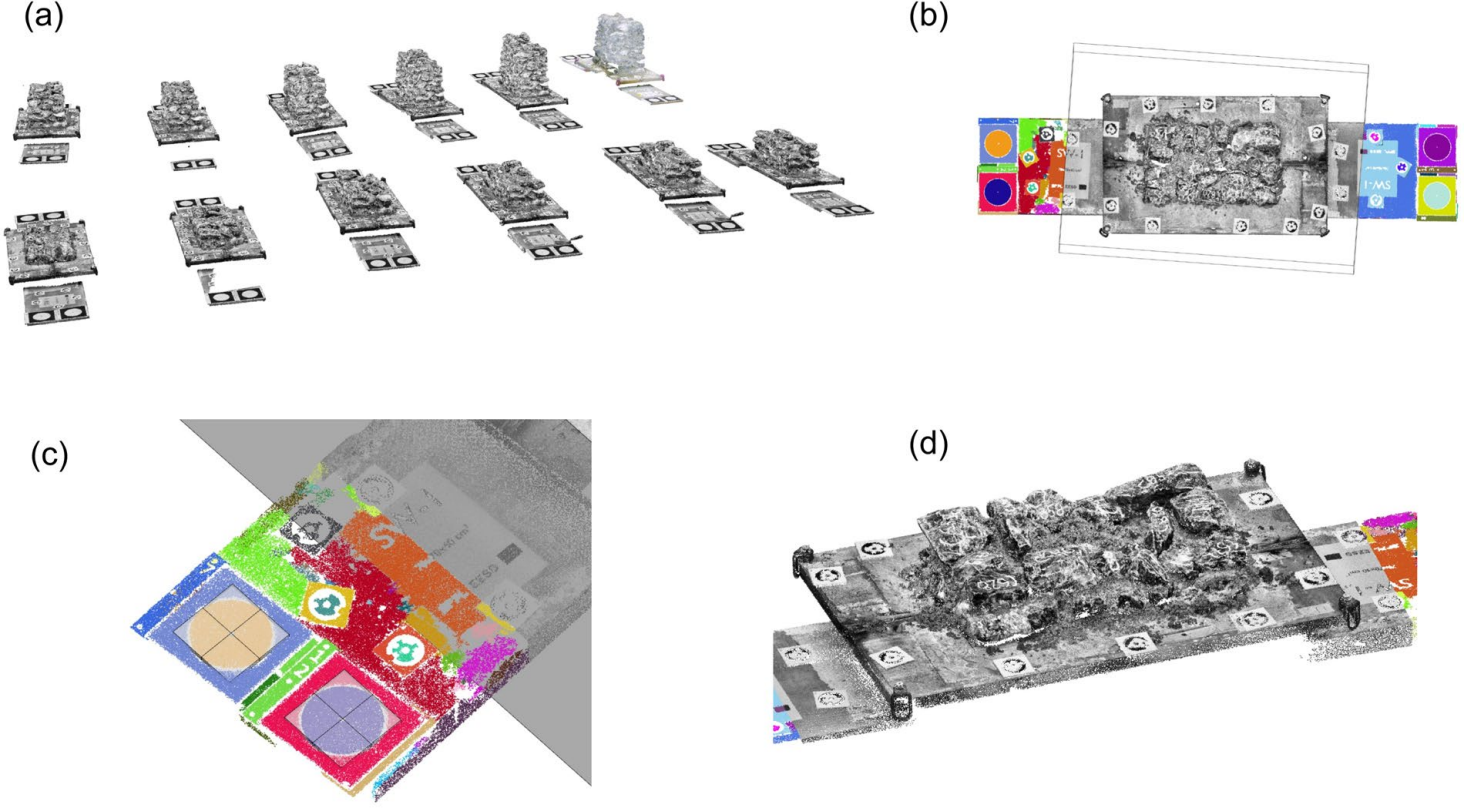
Digital reconstruction of walls. (**a**) All point clouds of the walls and targets obtained during the construction process were imported into the same working file. (**b**) Segmenting point clouds into two parts: the wall and the targets. Clustering the point cloud of the targets according to the RGB color. (**c**) Identifying the center of the targets. (**d**) Aligning the point clouds of the first two layers.
2. *Segmenting*: Segment each point cloud into two parts, one corresponding to the wall and one to the targets at the two sides of the wall. This procedure is performed manually by cropping the volume corresponding to the wall from the rest of the point cloud (Fig. 6b).
3. *Clustering of targets*: Cluster the points corresponding to each target into groups considering the RGB colors and a minimum neighboring distance (Fig. 6b).
4. *Target centroids*: Identify the center of each circular target as the volume centroid of a rectangular bounding box (Fig. 6c).
5. *Merging point clouds*: Merge the point clouds by applying plane-to-plane mapping. The procedure for each wall is as follows. For each wall layer scan, we used as a reference plane the one defined by the points at the centroids of three (out of the four) targets placed at the side of the walls. We computed the transformation matrix for mapping the reference plane of each scan to the reference plane of the scan of the first layer of the wall and used it to merge the two point clouds. (Fig. 6d).
6. *Point cloud down-sampling* (optional): Similar to the stones, the scans can be down-sampled to the requested resolution. In this work, we down-sampled the point clouds of the walls to a resolution of 2 *mm* only for post-processing purposes.
7. *Computing normals*: Compute the normals for each layer scan in the same way as they were computed for each stone. Computing the normals is necessary for the registration of each stone at its final position within the wall.
  In average, we needed 2 minutes to align each wall layer scan to the reference (first wall) scan. After aligning the scans, the following steps are implemented for each stone for the digital reconstruction of the wall:
8. *Identifying the stones of each layer*: Identify visually by means of their labels the stones belonging to each layer using the point clouds or the video and photos captured during wall construction.
9. *Placing stones*: Progressively place stones at their final position layer-by-layer. Starting from the first layer (bottom of the wall), import each stone belonging to that layer into the same working file as the wall scans. Manually place the stone close to its position within the wall (here we placed it so that we had an overlap of at least 10% with respect to its final position in the wall). Register its final position automatically using the same procedure as for registering the two halves of one stone (Fig. 7). Follow this procedure for all the stones belonging to the wall.

**Fig. 7:**
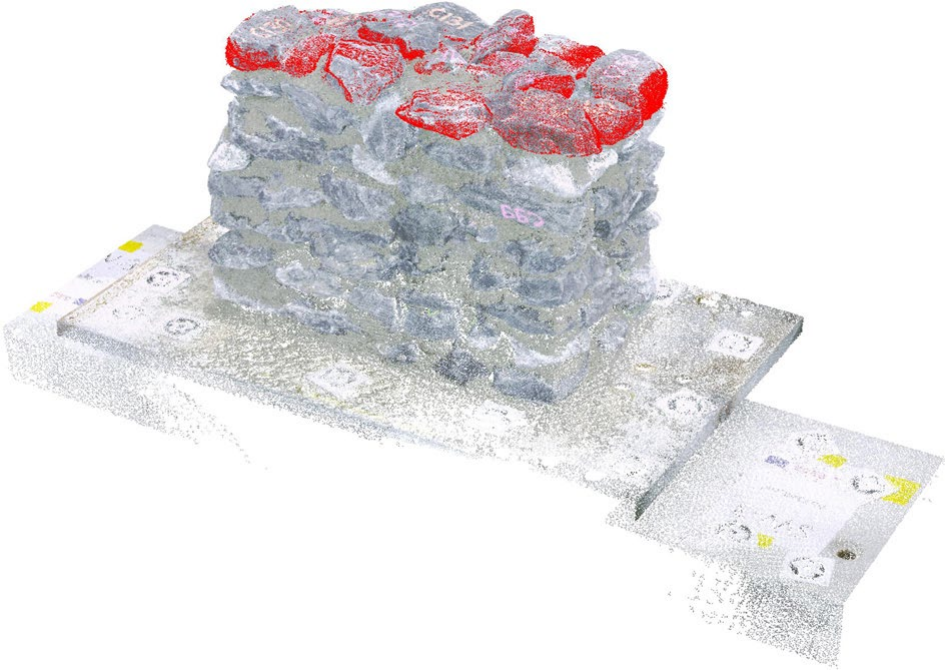
Supervised detection and reconstruction of stones in the wall layers. The scene is composed of the point cloud fragment of the as-built wall and the point cloud of the stones of the layer (red).
10. *Surface reconstruction* (optional). Depending on the use of the dataset, use the point cloud to reconstruct the stone surfaces. In this work, we performed the surface reconstruction for estimating the mortar volume by subtracting the volume of the stones from the wall volume and for checking collisions between stones. For this, a Poisson surface reconstruction method was used^41^.
  The placement of each stone, including the surface reconstruction, lasts in average 5 minutes.After all stones are placed within the wall volume, we check whether there are errors in the reconstruction process by searching for intersections between neighboring stones. This is done using a collision analysis algorithm, described in detail in the technical validation section. The following steps are applied iteratively until there are zero intersections between the stones:
11. *Collision analysis*: Check for intersections between stones using the collision analysis algorithm^42^ (see technical validation section).
12. *Correct stones position*: Adjust the position of the stones to eliminate the existing collisions. In this work, we changed the position of the stones manually applying translations to the stones of less than 5 *mm* and rotations of less than 10 degrees.

**Fig. 8:**
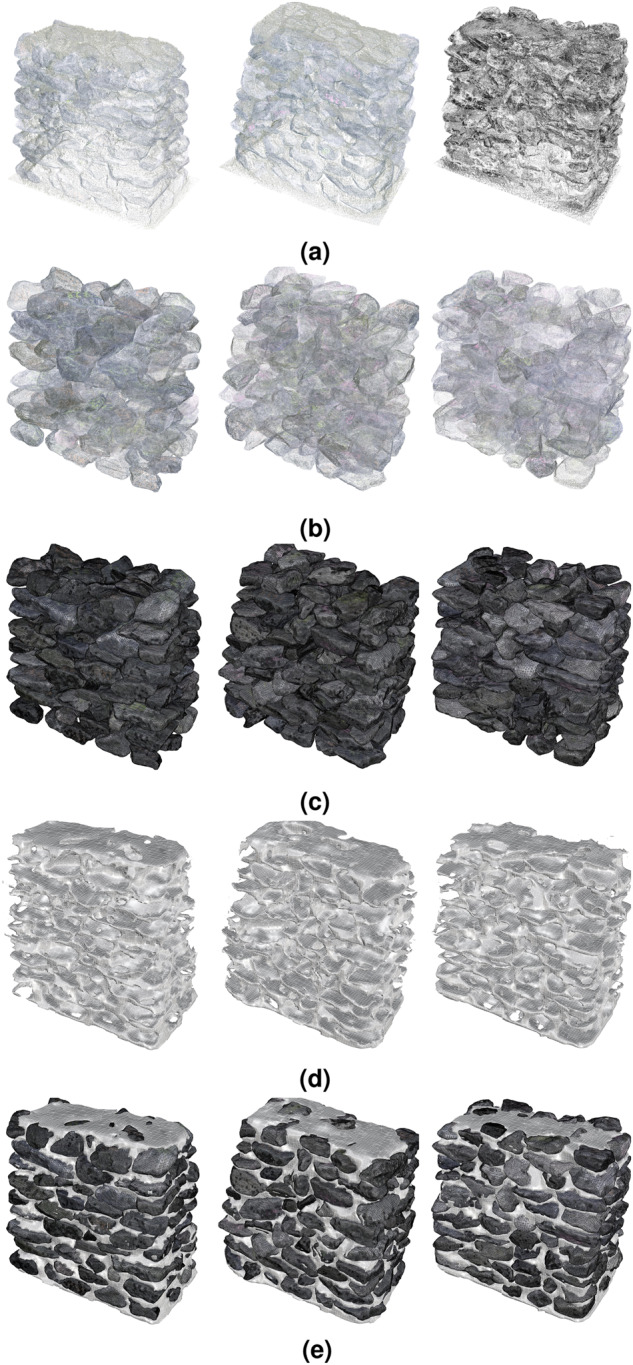
Overview of wall datasets: (**a**) Wall scans, (**b**) Point cloud of stones in their final position within the wall, (**c**) Surface meshes of the stones, (**d**) Surface mesh of the mortar, (**e**) Surface meshes of complete walls with stones and mortar.

**Fig. 9:**
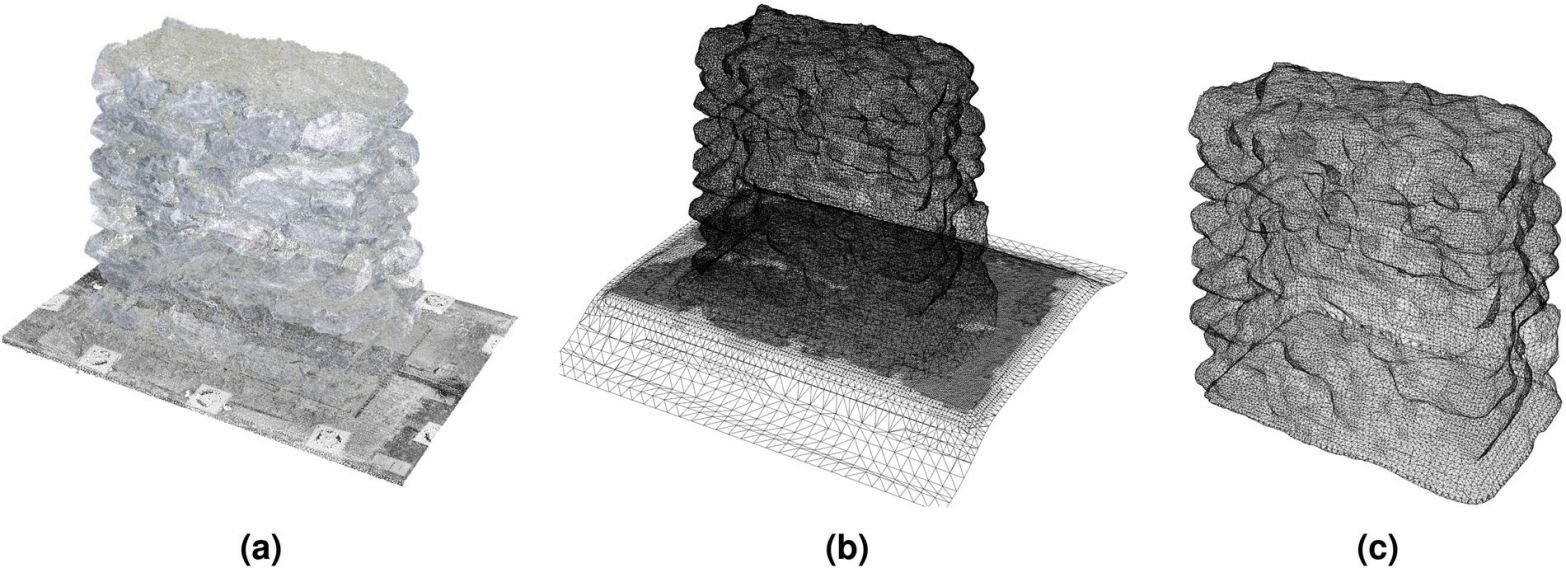
Meshing of the wall volume for generating the mortar volume: (**a**) Point clouds and (**b**) surface meshes of the plate without the wall (first scan) and of the final wall scan (final scan). (**c**) External mesh of the constructed wall obtained as the Boolean difference between the mesh of the final and first scans.

Figure 8a shows the final scan of the walls and Fig. 8b,c the point clouds and meshes of the stones in their final position, respectively. Once the wall is reconstructed, we obtained an approximate morphology and volume of the mortar by subtracting the volume of the stones from the volume of the entire wall. First, we generated a closed mesh of the total volume of the wall, computed as the Boolean mesh difference between the surface mesh of the final wall scan and the surface mesh of the plate at the base of the wall (first scan without the wall) (Fig. 9). For the subtraction, we opted for a voxel-based difference due to the high number of hypothetical Boolean operations between the above generated outer mesh and the mesh of each stone. The surface mesh of the wall and each stone were thus first defined as 5–*mm*-sized voxels (Fig. 9) before subtracting the volume of the stones from the total wall volume. The distinction between the mass occupied by the stones versus that of the mortar was then added to the model metadata to define the ratio between these two masonry components (Fig. 8d). Note that this computation gives an upper bound of the volume of the mortar. This is both due to the voxel-based mesh approximation and also to the fact that voids without mortar are expected to exist in several locations within the wall. Figure 8e illustrates the meshes of the final geometrical digital twins of each wall including stones and mortar.

Table 1 presents metadata related with the number of stones as well as mortar and wall volumes. For all the walls, we reconstructed and registered successfully all stones at their final position without any collisions. The metadata indicate that the size distribution of the stones within a wall varies between the three walls. All walls have similar wall and mortar volumes, but wall SW3 has 19% and 16% more stones than SW1 and SW2, respectively.

**Table 1:** Metadata obtained from the analysis of the geometrical digital twins of the three walls.

Wall ID	Number of Stones (-)	Mortar Volume (*m*^3^)	Wall Volume (*m*^3^)	Mortar/Wall Volume (%)
SW1	139	0.073	0.172	42.4
SW2	143	0.069	0.164	42.1
SW3	166	0.074	0.174	42.5

## Data Records

Tables 2, 3 present a summary of developed datasets, which are available on Zenodo (10.5281/zenodo.7093710)^43^.

**Table 2:** Description of the generated datasets for stones and walls.

dataset	Description	Type	Format	Size [GB]
Stones	Raw scans of stone batches	Point cloud	.e57	1.60
	Reconstructed stones per wall layer	Point cloud	.ply	0.69
Walls	Raw scans of wall layers	Point cloud	.e57	4.18
	Reconstructed walls	Point cloud	.ply	0.67
	Reconstructed walls - stones	Surface meshes	.ply	0.49
	Reconstructed walls - mortar	Surface meshes	.ply	0.03
	Reconstructed wall - stones, mortar, scans	Point clouds, meshes	.3dm	3.00
Total				10.66

**Table 3:** Description of the datasets for the collision analysis example.

Description	Type	Format	Size [GB]
Reconstructed wall example - stones, mortar, scans	Point clouds, meshes	.3dm	2.49
Input stones	Surface meshes	.ply	0.29
Output collisions	Surface meshes	.ply	0.005
Total			2.70

For the reconstruction of the stones, we provide raw scans of the stone batches (i.e. the unprocessed data from the laser scanning device). Only scans of bag C are provided, as the three walls were constructed exclusively with stones from this bag. The reconstructed stones are stored in folders that are labelled with the wall name and wall layer number (with increasing order from bottom to top).

For the reconstruction of the walls, we provide raw scans of the walls corresponding to each wall layer (Fig. 8a). For the reconstructed walls we provide the point clouds of all the stones at their final position (Fig. 8b), their corresponding surface meshes (Fig. 8c) and the surface mesh of the mortar (Fig. 8d). Figure 8e shows the meshes of the reconstructed walls (both stones and mortar).

We provide the .3dm file of each wall, which is the working file used in Rhinoceros3D for the wall reconstruction. Each .3dm file includes the point clouds and surface meshes of the reconstructed stones in their final position, the surface mesh of the mortar and the wall scans of each layer.

In addition to the reconstructed walls, we provide the .3dm working file of each wall and the input and output files used in the collision analysis examples described in the technical validation section (see Table 3).

## Technical Validation

### Collision algorithm

**Fig. 10:**
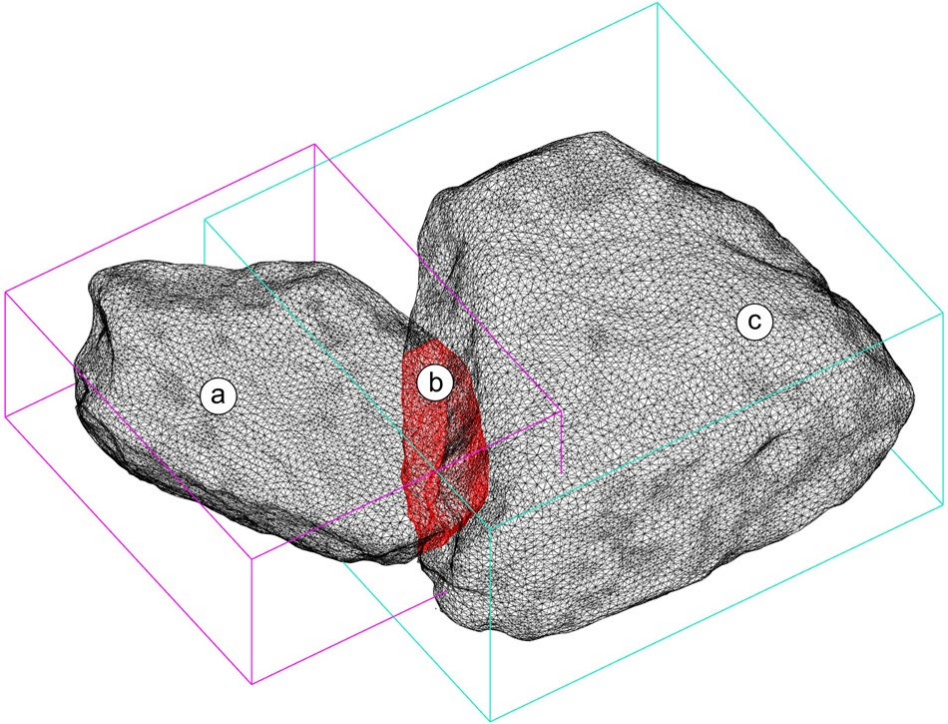
Collision between two stones. (**a**) Intersecting stone, (**b**) an intersection between the two colliding volumes, and (**c**) intersected stone. All stones are checked for collisions in each reconstructed wall.

To gauge the consistency of the developed digital reconstruction method in the absence of a ground truth model of the walls for comparison, we designed a collision analysis algorithm^42^. The purpose of the algorithm is to identify collisions between the meshes of the stones, which are provided as input. For each collision, the total volume of the two colliding stones is retrieved and compared with the collision volume (Fig. 10). The calculated ratio (termed as collision ratio) is thus equal to the volume of the collision divided by the total volume of the two colliding stones. We choose to compare the collision volume with the volume of the two stones instead of the volume of the whole wall in order to have a measure that is stone and wall-size independent. In this way, the collision ratio represents the accuracy of the relative location between two stones independent of the stone and wall size.

The output of the algorithm is the total number of collisions, the collision ratio, the volume of each of the colliding stones and a mesh of the surface of the colliding volume. As in the physical walls intersections between stones are not possible, the defined collision ratio can be used to evaluate the margin of error in the digital reconstruction, where the lower the ratio, the smaller the error.

From our experience with the preparation of this dataset, a collision may occur due to one or more of the following reasons:

(i) An imprecise stone registration due to the lack of sufficient resolution of the point cloud of a layer. As the accuracy of the registration algorithm depends on the number of points per stone, a sparse resolution of points per stone at the wall scan may result to a non-precise registration of the stone at its final position. A sparse resolution of points per stone could occurs due to errors during scanning (i.e., a non-homogeneous scan of the wall) or during construction (i.e. the partial cover of the top of a stone with mortar prior to a scan).
(ii) An imprecise stone reconstruction. This can occur either due to a sparse resolution of points per stone during the stone scanning procedure or an imprecise registration of two halves of a stone (item 6 of the section related with the digital reconstruction of stones). The imprecise stone reconstruction affects the geometry of the stone and thus the volume it occupies within the wall.
(iii) An already placed stone can move due to the construction process. From our experience, this can happen in at least two ways: due to the force applied by the mason during the placement of the stones of the following layers and due to the temporary removal and replacement of a stone. A change in the position of the external stones can be corrected by registering their position using the final scan of the wall. However, a change in the position of the internal stones cannot be retrieved once a new layer of stones has been placed above them. As mentioned below, this was the major source of collisions during the digital reconstruction of our walls.
(iv) The geometry of a stone could be slightly modified during storage or construction (i.e., accidental or manual fracture of parts of the stones during transfer or by the masons).

The first two errors could be minimized by automating the scanning procedure, and optimizing the registration procedure, respectively. Nevertheless, the movement of the already placed stones during the construction process cannot be easily addressed, as pressure must be applied on a newly placed stone to ensure its stability and proper adhesion with the mortar during the construction process. Changes in the geometry of the stones due to fracture could be also minimized by the handling of the stones by the masons or by automatizing the construction process.

### Application example of the collision algorithm

**Fig. 11:**
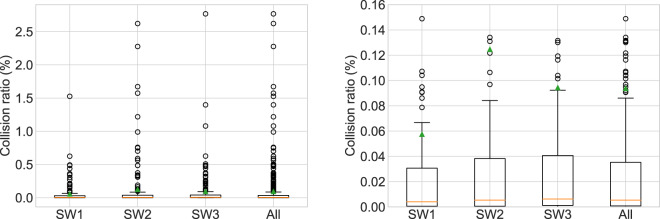
Box plots of the collision volume ratio for each collision per wall; refer to Table 4. For better interpretation, the plot in the left is repeated at the right with smaller upper bound of the y-axis. The orange line on each box indicates the median, the green triangle the mean value and the bottom and top edges of the box indicate the 25th (Q1 quartile) and 75th (Q3 quartile) percentiles, respectively. The whiskers extend from the box by 1.5x the inter-quartile range (IQR = Q3-Q1) and the outliers are plotted individually (black circles). Data refer to the first iteration of the collision analysis (step 11 of the digital wall reconstruction process).

Here, we present the results of the first collision analysis that we performed for each wall at the end of step 10 (surface reconstruction) in the digital reconstruction of walls procedure as described in the methods section. We calculated the mean, median, standard deviation, minimum and maximum ratios and report the values in Table 4. In addition, Fig. 11 displays the variation for each collision ratio in the form of box plots for each wall and for all walls together. All data (input meshes, output files and data processing scripts) are available at: 10.5281/zenodo.7093710^43^.

**Table 4:** Results of the application of the collision analysis algorithm for the three walls.

Wall ID (-)	Number of Collisions (-)	Mean Col. Ratio (%)	Median Col. Ratio (%)	SD Col. Ratio (%)	CoV Col. Ratio (%)	Min Col. Ratio (%)	Max Col. Ratio (%)
SW1	130	0.057	0.004	0.168	293	1.5e-10	1.527
SW2	150	0.125	0.005	0.375	301	6.1e-8	2.622
SW3	142	0.094	0.006	0.292	310	2.2E-7	2.768
All walls	422	0.094	0.005	0.296	316	1.5e-10	2.768

Data refer to the first iteration of the collision analysis (step 11 of the digital wall reconstruction process).

The analysis showed an average of 140 collisions per wall before any correction of the stone positions was performed. The average median ratio is 0.005%, while in all walls the 75% of the collisions have a collision ratio below 0.05%. This is a relatively low error considering the wall construction typology, where mortar joints are very thin or in-existent in some cases, and therefore contacts between stones exist. Outliers are above 0.08 and 0.10% for all walls and reach for some limited cases up to 2.8%. Despite the small number of outliers, their large values push the mean much further from the median, by at least an order of magnitude. The large scattering of the collision ratio is clearly shown by the high standard deviation and coefficient of variation.

**Fig. 12:**
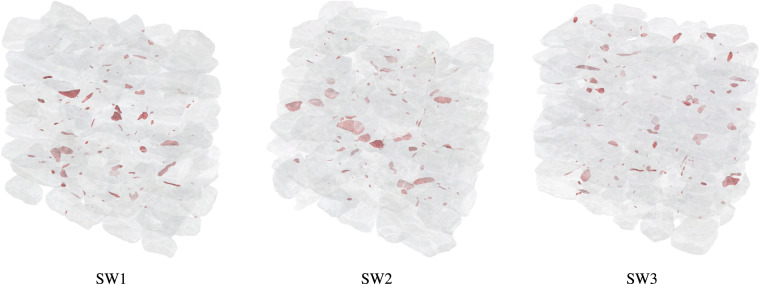
Visualisation of the colliding volumes (in red) within each wall at the first application of the collision analysis algorithm during the digital reconstruction process. Stone meshes are shown in transparent.

Figure 12 illustrates the colliding volumes within each wall. The majority of the collisions are related to small movements of the stones due to the pressure applied by the masons during the construction of the subsequent layers. Large collisions are mostly observed at internal stones, where the reconstruction difficulty is increased due to the previously described reasons.

By iteratively applying modifications to the positions of the stones and checking new collisions using the collision algorithm (steps 11 and 12 in the digital reconstruction methodology) we generated the geometrical digital twins of all the walls with zero collisions. As described, the position of the external stones was mainly modified by registering them to the final scan instead of the layer scan. Then, the position of the internal stones was modified such that they could fit between the external stones. In some singular cases, we had to reduce the volume of the stones in order to avoid collisions by scaling them. In those cases, a volume reduction of less than 2% was sufficient.

### Overview

Accurately investigating structural responses and validating numerical modeling techniques requires the precise geometrical modeling of a stone masonry wall or any other irregular composite material. Nevertheless, there are no accurate geometrical representations of irregular structural masonry walls in the literature. To address this research gap, this paper presents a dataset corresponding to the geometrical digital twins of three irregular three-leaf stone masonry walls constructed in the lab by experienced masons.

The presented pipeline is based on the acquisition of geometrical data about stones and walls through the use of portable laser scanning. The geometrical digital twins were developed through a supervised reconstruction. The following conclusions can be drawn from the implementation of the presented pipeline:

- The presented supervised reconstruction pipeline successfully produced geometrical digital twins for a complex aggregated structure–irregular stone masonry walls. The pipeline can be used for any size of walls and stones. In this work, we scanned stones above 50 *mm*, as smaller stones were used in rare occasions by the masons to fill voids between larger stones.
- Laser scanning provided highly accurate scaled point clouds that could be used directly in the geometrical twinning pipeline. Repeat scans were necessary on very few occasions due to an equipment failure that resulted in blurry scans. To avoid this problem during construction, two consecutive scans were made. In some cases, repeated scans proved to be important for avoiding data loss.
- Because the pipeline is supervised, the reconstructions of the walls and stone surfaces are prone to errors. A possible improvement that would increase its robustness would be a registration procedure based on the feature matching between points (or clusters of points). Features could include both geometrical and surface characteristics (i.e. RGB colors or greyscale gradients), which are obtained by the laser scanner. A similar approach was followed in^31^, but based on 2D features extracted from images.
- The creation of the stone dataset is highly labor intense at all stages: from batch scanning and stone reconstruction to the post-processing identification of each stone position in the wall (see Table 5). The most labor intensive part is related with the stone surface preparation (dust removal) and labelling. A more efficient strategy would involve an in-hand scanning technique and live-tracking of the stone during the construction phase. Another possibility would be to adopt an automatic registration based on matching both geometrical and surface texture features (i.e. RGB or grayscale information of the scanned points) as used in^31^.

**Table 5:** Average time per task in the laser-scanning pipeline.

Task	Time [minutes]
Surface preparation & labelling	2’ per stone
Stone scanning	10’ per batch
Wall scanning	3’ per *m*^2^
Stone reconstruction	5’ per stone
Wall scans alignment	2’ per wall layer
Stone registration to wall	5’ per stone

All reported times consider the implementation of the pipeline by a single person.
- The collision analysis is a useful tool for checking the accuracy of the procedure and correcting errors in the reconstruction process.

## Usage Notes

To the best of our knowledge, this is the first dataset with a realistic representation of the micro-structure of as-built irregular stone masonry walls with mortar. Since all of the walls have been tested under quasi-static shear compression loading (related dataset to be published soon), this data offers a unique opportunity for bench-marking existing numerical modelling techniques based on the wall’s microstructure (e.g. FEM micro-modelling, Discrete Element modelling) against the experimental results. For this, we have made available the final geometrical digital twin of each wall without collisions on Zenodo^43^. We also provide raw scans of walls and stones, which can be used for the implementation of the presented pipeline^43^.

## Data Availability

The pipeline is based on the use of Rhinoceros3D^35^ and the Cockroach plugin which is available at https://ibois-epfl.github.io/Cockroach-documentation/^36^. A tutorial for the implementation of the pipeline is freely available online^37^. In this work, we used Rhinoceros3D Version 7 SR29. The collision analysis algorithm is available at: https://github.com/ibois-epfl/collide^42^. The Python script used for the analysis of the collision data is available at: 10.5281/zenodo.7093710^43^.
